# Determinants of Adherence to a Gluten-Free Diet in Children with Celiac Disease and the Influence of the Methods Used to Assess It

**DOI:** 10.3390/nu15112455

**Published:** 2023-05-24

**Authors:** Alice Monzani, Silvia Marcolin, Sara Giorda, Francesco Epis, Maristella Babral, Kevin Valentino, Lorenza Scotti, Enrico Felici, Ivana Rabbone

**Affiliations:** 1 Division of Pediatrics, Department of Health Sciences, Università del Piemonte Orientale, 28100 Novara, Italy; 20026340@studenti.uniupo.it (F.E.); 20022157@studenti.uniupo.it (M.B.); kevin.valentino.93@gmail.com (K.V.); ivana.rabbone@uniupo.it (I.R.); 2 Italian Celiac Association, Piedmont Section, 10136 Turin, Italy; s.marcolin@aicpiemonte.it (S.M.); saragiorda.91@gmail.com (S.G.); 3 Department of Translational Medicine, Università del Piemonte Orientale, 28100 Novara, Italy; lorenza.scotti@uniupo.it; 4 Pediatric and Pediatric Emergency Unit, Children Hospital, AO SS Antonio e Biagio e C. Arrigo, 15121 Alessandria, Italy; enrico.felici@ospedale.al.it

**Keywords:** adherence, celiac disease, children, dietitian, gluten-free diet

## Abstract

Lifelong adherence to a gluten-free diet (GFD) is the cornerstone of management of celiac disease (CD), but adhering to a GFD can be hard. Although several factors are positively associated with adherence of pediatric CD patients to a GFD, it is unknown whether these are influenced by variability caused by the specific tool used to assess adherence to a GFD. Here, we aimed to evaluate how individual patient characteristics and dietary counselling by a trained dietitian influence adherence to a GFD in children with CD, as assessed by two validated questionnaires: the Biagi questionnaire and the Leffler short questionnaire adapted for pediatric patients. Some 139 children and adolescents were recruited in a cross-sectional, multicenter study. Concordance between the two questionnaires in defining adherence was fair (weighted Cohen’s kappa coefficient 0.39, 95%CI 0.19–0.60). Upon regression analysis, having a cohabiting family member with CD, being of Italian origin, and receiving specialized dietary counselling during follow-up were found to positively influence stricter adherence to a GFD for children with CD. Neither questionnaire detected a significant relationship between adherence to a GFD and the presence of symptoms after gluten ingestion. This study provides important new data on the factors influencing GFD adherence in the pediatric population, and highlights the importance of dietician input and overcoming language and cultural barriers when educating patients.

## 1. Introduction

Lifelong adherence to a gluten-free diet (GFD) is the cornerstone of management of celiac disease (CD) in both adults and children [1]. Strict and lifelong elimination of gluten from the diet is strongly recommended, not only for symptom control, but also to decrease the risk of complications [2]. However, strict adherence to a GFD can be hard, as it requires total avoidance of all products containing wheat, barley, and rye [3], which are essential staples in many parts of the world. As alternatives, uncontaminated natural gluten-free foods may not always be available, and commercially prepared substitutes tend to be expensive—and sometimes less palatable—than their gluten-containing counterparts [3]. Thus, strict adherence to a GFD can be challenging, as it can be compromised by unnoticed gluten consumption, cross-contamination, or social pressure when eating out, even more so when symptoms are non-specific or absent after accidental or voluntary gluten ingestion [4]. The reported rates of strict adherence to a GFD are between 42% and 91% in adults with CD [5], and between 23% and 98% in children and adolescents with CD [2].

At least some of the variability in reported adherence might be due to the wide range of tools used to assess adherence to a GFD. Even though most gastrointestinal societies recommend routine assessment of GFD adherence [6,7,8,9], there is currently no standardized method to perform it. The assessment of duodenal biopsies, which is considered the gold standard, is not feasible for routine CD follow-up in every case, due to these biopsies’ invasiveness, relative risk, and high cost, especially in children. Gastroenterologists frequently use a combination of self- and parent-reported adherence, the presence of symptoms, dietary assessment by a trained dietitian, and biomarkers (both in the blood and in urine/stool samples), even though it is accepted that these methods are limited for accurately detecting gluten transgressions and histologic recovery [9,10,11,12].

Nevertheless, several studies have highlighted several factors that are positively associated with adherence of pediatric CD patients to a GFD: having a first-degree relative with celiac disease [13], the presence of gastrointestinal symptoms [14], good parental knowledge about CD [2], and being a member of a CD patient society [2].

Here, we aimed to evaluate how individual patient characteristics and dietary counselling by a trained dietitian influence adherence to a GFD in children with CD, as assessed by two validated questionnaires and serologic testing (anti-transglutaminase immunoglobulin A, TGA-IgA).

## 2. Materials and Methods

### 2.1. Patient Selection

This was a cross-sectional multicenter study of consecutively recruited children and adolescents undergoing follow-up for CD at the Pediatric Gastroenterology Outpatient Clinic of Maggiore della Carità University Hospital in Novara, Piedmont, Italy, or at the Pediatric Gastroenterology Outpatient Clinic of Santi Antonio e Biagio e Cesare Arrigo Children Hospital in Alessandria, Piedmont, Italy, between August and December 2022.

The inclusion criteria were (i) celiac disease diagnosed according to ESPHGAN guidelines and at least one year prior to enrolment [15]; (ii) aged between 2 and 17 years of age; (iii) informed consent from the child’s parent and the child, as appropriate; and (iv) availability of TGA-IgA values at diagnosis and just before the follow-up visit (a time frame of one month was considered acceptable). The exclusion criteria were (i) an insurmountable language barrier; (ii) comorbid psychiatric conditions; and (iii) IgA deficiency.

### 2.2. Study Design and Methods

During the annual follow-up visit, patients and/or family members were asked to complete two questionnaires assessing adherence to a GFD, the Biagi questionnaire [16] and the Leffler short questionnaire adapted for pediatric patients [17,18], in a face-to-face interview. Specifically, in patients younger than 12 years of age, the parent or caregiver answered questions, taking into consideration the patient’s opinion, while for patients 12 years or older, the patient themself answered, with support given by family members where needed.

The Biagi questionnaire consists of four simple questions that classify patients into three groups of GFD adherence: a score of 0–1 denotes poor adherence; a score of 2 denotes adherence characterized by major errors that need correction; and a score of 3–4 denotes excellent adherence. The modified Leffler questionnaire consists of eight questions that investigate the patient’s voluntary and involuntary exposure to gluten, GFD-specific knowledge, self-efficacy, and individual disease perception. Each answer is given a score of 0–3. According to the total, patients are classified into three groups of GFD adherence: a score of ≤2 points is excellent; a score of 3–6 points is fair; and a score ≥ 7 is low.

At the same time as administering the questionnaires, the result of a recent TGA-IgA determination was collected (a result within one month was considered acceptable). As not all patients had their TGA-IgA levels measured in the same laboratory, TGA-IgA values were expressed as the number of times the upper limit of normal (×ULN) for each laboratory. This value at the time of the interview was compared with the initial value at diagnosis to calculate the reduction over time.

For each patient, the following data were collected: sex, country of origin, age at CD diagnosis, modality of CD diagnosis (biopsy sparing or not), time since CD diagnosis at interview, presence of CD-related symptoms before CD diagnosis, presence of one or more relatives/cohabitants with CD, reported symptoms on occasions of accidental or voluntary gluten consumption when on a GFD, and whether or not they had received a nutritional counselling intervention at the time of diagnosis and/or during follow-up by dietetic staff specifically trained in GFD.

The study was conducted in adherence with the regulations established by the local ethics committee, the Declaration of Helsinki, and Good Clinical Practice guidelines. Informed written consent was obtained from all subjects’ parents and from the patients, where appropriate, and the local ethics committee (Comitato Etico Interaziendale, Novara, Italy) approved the study protocol.

### 2.3. Statistical Analysis

Descriptive statistics were used to summarize the main characteristics of the subjects. Categorical variables are reported as absolute frequencies and percentages, while numerical variables are reported as means and standard deviations (SD) or medians and interquartile ranges (IQR) if not normally distributed according to the Shapiro-Wilk test and QQ plots. The concordance between the Biagi and modified Leffler scores in classifying subjects as adherent to GFD was evaluated using a weighted Cohen’s kappa and the corresponding 95% confidence intervals (95%CI). For this analysis, scores were classified into the three GFD adherence groups, as previously reported and described above. Univariable Poisson regression models with robust variance were used to estimate the relative risk (RR), the corresponding 95%CI for associations between patient characteristics, and the probability of being adherent to GFD, as evaluated by both the Biagi and modified Leffler scores. For this analysis, both Biagi and modified Leffler scores were divided into two classes of adherence: optimal (Biagi score ≥ 3, Leffler score ≤ 2) and suboptimal (Biagi score < 3, Leffler score > 2). Finally, linear regression models were applied to evaluate the relationship between patient characteristics and TGA-IgA × ULN measured at the follow-up visit. For this latter analysis, outlier observations were removed to meet the assumptions of the linear model. All analyses were also performed, adjusting for time since diagnosis to account for different disease durations.

## 3. Results

### 3.1. Patient Characteristics

Some 139 pediatric patients with CD were recruited (87 females and 52 males), with a mean age at diagnosis of 6.9 ± 3.5 years; 127 subjects (91.4%) were Italian. The median disease duration was 2.37 years (IQR 1.10–6.08).

Before diagnosis, 123 subjects (88.5%) reported experiencing at least one CD-related symptom, while 16 (11.5%) did not complain of any symptoms and were screened due to a positive family history or other associated autoimmune disease. Among the symptoms reported at diagnosis, the most frequent were recurrent abdominal pain, affecting 64/139 patients (46%) and over half of the 123 symptomatic patients (52%); impaired growth, affecting 28 patients (20.1%); diarrhea in 19 subjects (13.7%), constipation in 10 subjects; dermatitis in 8 patients (5.8%); and recurrent oral aphthosis in 7 patients (5%).

The diagnosis was made via esophagogastroduodenoscopy (EGDS) in 55 subjects (39.6%), while 84 (60.4%) had a biopsy-sparing diagnosis. Median (IQR) TG-IgA levels × ULN were 22.2 (10.2–125.7) at diagnosis and 0.56 (0.21–1.52) at follow-up, representing a median relative reduction of 97.6% (93.6–99.3%). The median time from diagnosis was 2.1 years (IQR: 1.10–6.08).

Some 29 of the 139 patients (20.9%) had a cohabiting first-degree relative with CD (mother for 10 children, father for 7 children, one sibling for 12 children), while 12 subjects (8.6%) had a non-cohabiting relative (cousin, uncle/aunt, grandparent) with CD.

Regarding access to a registered dietitian, 32 patients (23%) received dietary counselling from an expert dietitian at the time of diagnosis, whereas 107 (77%) received GFD education from a pediatric gastroenterologist. During follow-up, 84 patients (60.4%) received counselling from a trained dietitian, while 55 (39.6%) did not. Some 22 subjects (15.8%) received dietary counselling both at diagnosis and during follow-up.

Regarding sensitivity to purposeful or accidental gluten intake when following a GFD, 26 subjects (18.7%) reported symptoms after gluten ingestion (namely, abdominal pain, vomiting, diarrhea), 24 patients (17.3%) reported no symptoms after gluten consumption, and 89 patients (64%) were unable to declare if they were symptomatic to possible cross-contamination, because they apparently had not experienced any gluten ingestion since starting their GFD.

### 3.2. GFD Adherence Assessed by Questionnaires

According to the Biagi questionnaire, most patients (91.4%) were strictly adherent to their GFD (scores of 3 or 4), and only 8.6% of patients had poor adherence (scores of 0–1). No patients were classified as adherent but committing errors, with a score of 2 (Table 1).

According to the modified Leffler questionnaire, 81.3% had excellent adherence (score 0–2), 13.2% had fair adherence (score of 3–6), and 5.5% had low adherence to the GFD (score of 7–10) (Table 2).

The Biagi and modified Leffler questionnaires showed only fair agreement, with a weighted Cohen’s kappa coefficient of 0.39 (95%CI 0.19–0.60).

### 3.3. Determinants of GFD Adherence

The influence of individual factors and dietetic intervention on GFD adherence as assessed by Biagi scoring is shown in Table 3. Cohabiting with a family member with CD increased the probability of optimal adherence by 12% compared with those without family members with CD, even after adjusting for disease duration. Being of Italian origin and having symptoms after gluten ingestion when on a GFD tended to be related to better adherence, but not significantly.

The influence of individual factors and dietary intervention on GFD adherence as assessed by the modified Leffler questionnaire is shown in Table 4. Being of non-Italian origin reduced the probability of optimal adherence by 51%, also after adjusting for disease duration, and receiving counselling from a trained dietitian during follow-up increased it by 22% after adjusting for disease duration.

When analyzing the potential influence of the different factors on TGA-IgA levels at follow-up, after adjusting for disease duration, children with an EGDS diagnosis had, on average, 0.76-times lower TGA-IgA × ULN values than those with a biopsy-sparing diagnosis. For an increase of one year of age and disease duration, the TGA-IgA × ULN values decreased by 0.91 and 0.08, respectively, while for an increase of one unit in TGA-IgA × ULN at diagnosis, the values of TGA-IgA × ULN at follow-up visit increased by 0.001. There was no relationship with either the Biagi or modified Leffler scores (Table 5).

## 4. Discussion

In this study, we aimed to assess, using different instruments, GFD adherence in a cohort of pediatric patients with CD, and to identify possible associated factors. The main result of our analysis was that the classification of adherence varies according to the questionnaire (from 91.4% of strictly adherent patients with Biagi scoring to 81.3% with modified Leffler scoring), with only fair concordance between the Biagi and modified Leffler scores in classifying subjects as strictly adherent to GFD. Neither instrument was correlated with TGA-IgA levels at the time of interview.

We can hypothesize that the Biagi questionnaire found higher adherence to GFD as it only has four questions, is easy to understand, and allows only two answers (yes or no). The Biagi score can be regarded as quite restrictive, as answering “yes” to the first question (Do you eat gluten intentionally?) already classifies the patient as poorly adherent. However, the lower prevalence of excellent adherence detected using the modified Leffler’s questionnaire may be due to multiple factors. First, there are more questions in this instrument that may be difficult to understand, and each allows four answers. For example, the answer to the first question (“What is your average gluten intake?”) could make a big difference to the adherence outcome as, to be considered strict, the GFD should never allow voluntary gluten intake. However, for this question, the answer “less than three times a year” is still regarded as excellent adherence, so it may not distinguish patients who never transgress from the GFD from ones who voluntarily take gluten, albeit occasionally. Moreover, both questionnaires do not investigate the type of gluten consumption, not discriminating between the deliberate consumption of a wheat-based product, such as bread or pizza, and a food with a “may contain” statement. Such an issue could be crucial in influencing the serology and biopsy assessment of dietary adherence [19,20,21,22]. Regarding the amount of voluntary gluten consumption in addition to frequency, only the Biagi questionnaire focuses on the amount of intake, distinguishing between “a normal portion” and “just a taste”. Furthermore, the modified Leffler questionnaire also includes a question referring to the patient’s self-perceived health status (“Do you consider yourself ill?”), with a negative answer suggestive of better adherence to a GFD. This interpretation is questionable, because it is conceivable that self-perceived illness could imply a higher level of attention to cross-contamination, if considered potentially harmful by the patient.

Our results suggest that the assessment of adherence to a GFD in pediatric patients should be multidisciplinary, because the exclusive use of questionnaires designed to evaluate adherence may be affected by bias. Furthermore, children or adolescents visiting clinic with their parents might not tell the truth when the questionnaires are administered, for fear of negative judgement. Therefore, it could be inferred that standardized questionnaires may be used to give an overall impression of the degree of adherence to a GFD. Still, an experienced dietitian should be assessing CD patients and asking more in-depth, directed questions that are tailored to the individual subject.

The variables associated with adherence to a GFD differed according to the tool used. Having a cohabiting family member sharing the same diagnosis of CD was a favorable determinant of strict adherence assessed by the Biagi questionnaire, as previously reported by Metha et al. [13]. It is likely that when more than one family member has a diagnosis of CD, strict adherence to a GFD is easier at home and also when eating out, at least for younger children who share most of their time with their parents. When assessed using the modified Leffler questionnaire, CD adherence was most positively influenced by Italian origin and specialized dietician counselling during follow-up. Although patients with a strong linguistic barrier were excluded from the study, it is possible that suboptimal fluency in Italian would have prevented full comprehension of the principles of GFD explained at diagnosis, making non-Italian patients more prone to dietary errors. Translation services at this critical point of management may be advisable if even the linguistic barrier is slight. According to the modified Leffler score, consultation with a dietician seemed to be relevant to GFD adherence only during follow-up and not at diagnosis. This might be because patients not receiving dietician consultations at diagnosis were instructed by a pediatric gastroenterologist on the principles of GFD, possibly smoothing the differences in GFD adherence between patients either receiving or not a specialized dietitian consultation at diagnosis. It is understood that patients should be referred to a trained dietitian until diagnosis. Nevertheless, our result highlights the crucial role played by trained dietitians during follow-up in reinforcing the importance of a GFD and correcting potential errors. Overall, even with the limitation of the low concordance between the questionnaires, having a cohabiting family member with CD, being fluent in the local language, and receiving specialized dietary counselling during follow-up seem to be predictors of stricter adherence to a GFD for children with CD.

Neither questionnaire detected a significant relationship between adherence to a GFD and the presence of symptoms after gluten ingestion. Cross-contamination may be an issue for patients with CD, as even small amounts of gluten can trigger an immune response and possibly cause symptoms. It is important for people with CD to be aware of potential sources of contamination, such as shared cooking utensils, cross-contamination with gluten-containing foods, and non-food items that may contain gluten. Although our data suggest that a significant percentage of patients had apparently never experienced gluten contamination, complete avoidance of gluten is unlikely, and it is just as likely they were asymptomatic to accidental gluten exposure. Even in the absence of gluten-triggered symptoms, it is important that healthcare providers discuss this issue with all CD patients and provide education and support to avoid potential sources of contamination. On the other hand, it should be considered that the presence of symptoms in a CD patient on a GFD does not automatically mean that gluten is being eaten.

As expected, TG-IgA levels at interview were directly associated with a biopsy-sparing diagnosis, as this mode of diagnosis is only allowed in the presence of TGA-IgA > 10 × ULN, a shorter disease duration, and higher levels at diagnosis. No relationship was found with adherence scores, suggesting that TGA-IgA is not an ideal tool, not only for assessing histologic recovery [11,12], but also for discriminating between strictly and poorly compliant patients [13].

## 5. Conclusions

In conclusion, having a cohabiting family member with CD, being fluent in the local language, and receiving specialized dietary counselling during follow-up seem to positively influence stricter adherence to a GFD for children with CD.

It is widely recognized that currently available biomarkers and questionnaires do not reflect histologic recovery. Nevertheless, in CD, this is probably not the (only) therapeutic goal; rather, the goal is to avoid exposure to gluten as an immunological trigger with a different individual threshold not necessarily evidenced by symptom persistence or appearance. It is from this perspective that the advent of new non-invasive biomarkers that can detect harmful exposure to gluten would be desirable.

## Figures and Tables

**Table 1:** Distribution of adherence scores according to the Biagi questionnaire.

Biagi Score	N° of Patients	%	Level of GFD Adherence
0	4	2.87%	Poor
1	8	5.75%	
2	0	-	Fair
3	98	70.50%	Excellent
4	29	20.86%	

**Table 2:** Distribution of adherence scores according to the modified Leffler questionnaire.

Modified Leffler Score	N° of Patients	%	Level of GFD Adherence
0	39	28.05%	Excellent
1	35	25.17%	
2	39	28.05%	
3	12	8.63%	Fair
4	2	1.43%	
5	2	1.43%	
6	3	2.15%	
7	2	1.43%	Low
8	3	2.15%	
9	1	0.71%	
10	1	0.71%	

**Table 3:** Distribution of patient characteristics according to GFD adherence assessed by Biagi scoring, with relative risks (RR), 95%CI, and *p*-values derived from univariable Poisson regression models and adjusted by disease duration (aRR).

	GFD Adherence by Biagi Score				
	Suboptimal *n* = 12	Optimal *n* = 127	*p*-Value	RR (95%CI)	aRR * (95%CI)
Country, *n* (%)					
Italy	8 (66.67)	119 (93.70)	0.0975	1	1
Outside Italy	4 (33.33)	8 (6.30)		0.71 (0.48–1.06)	0.71 (0.48–1.07)
Sex, *n* (%)					
Male	8 (66.67)	48 (37.80)	0.7549	1	1
Female	4 (33.33)	79 (62.20)		0.98 (0.89–1.09)	0.98 (0.89–1.09)
Symptoms at diagnosis, *n* (%)					
No	2 (16.67)	14 (11.02)	0.6198	1	1
Yes	10 (83.33)	113 (88.98)		1.05 (0.87–1.27)	1.05 (0.86–1.27)
Cohabiting with CD, *n* (%)					
No	12 (100.00)	98 (77.17)	0.0005	1	1
Yes	0 (0.00)	29 (22.83)		1.12 (1.05–1.2)	1.12 (1.05–1.2)
Diagnosis by EGDS, *n* (%)					
No	8 (6.67)	76 (59.84)	0.6349	1	1
Yes	4 (33.33)	51 (40.16)		1.02 (0.93–1.13)	1.02 (0.9–1.16)
Dietician consultation at diagnosis, *n* (%)					
No	10 (83.33)	97 (76.38)	0.5430	1	1
Yes	2 (16.67)	30 (23.62)		1.03 (0.93–1.15)	1.05 (0.92–1.21)
Dietician consultation at follow-up, *n* (%)					
No	6 (50.00)	49 (38.58)	0.4601	1	1
Yes	6 (50.00)	78 (61.42)		1.04 (0.93–1.16)	1.04 (0.94–1.16)
Symptoms after gluten ingestion, *n* (%)					
No	5 (83.33)	19 (43.18)	0.0821	1	1
Yes	1 (16.67)	25 (56.82)		1.21 (0.98–1.51)	1.21 (0.98–1.5)
Missing	6	83			
Age, mean (SD)	7.83 (4.69)	6.25 (3.40)	0.2538	0.99 (0.97–1.01)	0.99 (0.97–1.01)
Disease duration (yrs), median (IQR)	1.63 (1.09–4.98)	4.09 (1.1–6.08)	0.7844		
TGA-IgA × ULN at diagnosis, median (IQR)	15.70 (8.69–119.0)	24.03 (10.18–125.7)	0.1383		
Relative difference TGA-IgA × ULN at diagnosis–follow-up visit, median (IQR)	95.72 (94.23–98.37)	97.93 (93.16–99.29)	0.7234		

* adjusted for disease duration.

**Table 4:** Distribution of patient characteristics according to GFD adherence as assessed by the modified Leffler score, with relative risks (RR), 95%CI, and *p*-values derived from univariable Poisson regression models and adjusted by disease duration (aRR).

	GFD Adherence by Modified Leffler Score				
	Suboptimal *n* = 26	Optimal *n* = 113	*p*-Value	RR (95%CI)	aRR * (95%CI)
Country, *n* (%)					
Italy	19 (73.08)	108 (95.58)	0.0379	1	1
Outside Italy	7 (26.92)	5 (4.42)		0.49 (0.25–0.96)	0.49 (0.25–0.96)
Sex, *n* (%)					
Male	11 (42.31)	41 (36.28)	0.5774	1	1
Female	15 (57.69)	72 (63.72)		1.05 (0.89–1.24)	1.05 (0.89–1.24)
Symptoms at diagnosis, *n* (%)					
No	4 (15.38)	12 (10.62)	0.5467	1	1
Yes	22 (84.62)	101 (89.38)		1.09 (0.82–1.47)	1.1 (0.81–1.48)
Cohabiting with CD, *n* (%)					
No	23 (88.46)	87 (76.99)	0.1166	1	1
Yes	3 (11.54)	26 (23.01)		1.13 (0.97–1.33)	1.13 (0.97–1.32)
Diagnosis by EGDS, *n* (%)					
No	15 (57.69)	69 (61.06)	0.7543	1	1
Yes	11 (42.31)	44 (38.94)		0.97 (0.83–1.15)	0.97 (0.8–1.17)
Dietician consultation at diagnosis, *n* (%)					
No	21 (80.77)	86 (76.11)	0.5886	1	1
Yes	5 (19.23)	27 (23.89)		1.05 (0.88–1.25)	1.06 (0.86–1.3)
Dietician consultation at follow-up, *n* (%)					
No	15 (57.69)	40 (35.40)	0.0550	1	1
Yes	11 (42.31)	73 (64.60)		1.19 (1–1.43)	1.22 (1.01–1.47)
Symptoms after gluten ingestion, *n* (%)					
No	2 (33.33)	22 (50.00)	0.4408	1	1
Yes	4 (66.67)	22 (50.00)		0.92 (0.75–1.13)	0.92 (0.75–1.12)
Missing	20	69			
Age, mean (SD)	7.54 (4.50)	6.12 (3.24)	0.1292	0.98 (0.95–1.01)	0.98 (0.95–1)
Disease duration (yrs), median (IQR)	1.64 (1.10–5.80)	2.61 (1.10–6.08)	0.9837		
TGA-IgA × ULN at diagnosis, median (IQR)	31.0 (12.2–203.0)	21.29 (9.61–122.85)	0.5748		
Relative difference TGA-IgA × ULN at diagnosis–follow-up visit, median (IQR)	96.62 (89.50–98.52)	97.92 (93.55–99.31)	0.3309		

* adjusted for disease duration.

**Table 5:** Values of the model parameter (beta), standard error (se), and *p*-values for the relationship between patient characteristics and TGA-IgA × ULN at follow-up visit, derived from univariable linear regression modeling and adjusted for disease duration.

	TGA-IgA × ULN at Follow-Up Visit			
	Univariable		Adjusted for Disease Duration	
	Beta (se)	*p*-Value	Beta (se)	*p*-Value
Country				
Outside Italy vs. Italy	0.206 (0.328)	0.5313	0.093 (0.315)	0.7684
Sex				
Female vs. male	0.325 (0.174)	0.0651	0.306 (0.166)	0.0685
Symptoms at diagnosis				
Yes vs. No	−0.078 (0.278)	0.7810	0.058 (0.268)	0.8303
Cohabiting with CD				
Yes vs. No	−0.118 (0.209)	0.5744	0.158 (0.19)	0.6607
Diagnosis by EGDS				
Yes vs. No	−0.872 (0.154)	<0.0001	−0.756 (0.168)	<0.0001
Dietary consultation at diagnosis				
Yes vs. No	0.306 (0.214)	0.1563	−0.007 (0.227)	0.9756
Dietary consultation at follow-up				
Yes vs. No	−0.105 (0.177)	0.5559	0.092 (0.178)	0.6041
Symptoms after gluten ingestion				
Yes vs. No	0.376 (0.277)	0.1822	0.364 (0.272)	0.1883
Age (years)	−0.066 (0.025)	0.0092	−0.908 (0.200)	<0.0001
Disease duration (yrs)	−0.077 (0.021)	0.0004		
TGA-IgA × ULN at diagnosis	0.001 (0.000)	<0.0001	0.001 (0.000)	<0.0001
Relative difference TGA-IgA × ULN at diagnosis–follow-up visit	−0.002 (0.011)	0.8607	0.006 (0.620)	0.5380
Biagi score				
Optimal vs. Suboptimal	0.227 (0.328)	0.4906	0.261 (0.313)	0.4055
Modified Leffler score				
Optimal vs. Suboptimal	0.074 (0.219)	0.7354	0.094 (0.209)	0.6537

## Data Availability

The data presented in this study are available on request from the corresponding author.
